# Definitive Confirmation of Erythropoietic Protoporphyria via Re-biopsy Three Years After Initial Liver Biopsy at Age 15

**DOI:** 10.7759/cureus.38017

**Published:** 2023-04-23

**Authors:** Yuko Miyakami, Takeo Minamikawa, Hirohisa Ogawa, Mayuko Ichimura-Shimizu, Kohichi Tsuneyama

**Affiliations:** 1 Pathology and Laboratory Medicine, Graduate School of Biomedical Sciences, University of Tokushima, Tokushima, JPN; 2 Engineering, Laboratory for Advanced Photonic Science and Technology, University of Tokushima, Tokushima, JPN

**Keywords:** children, fluorescence microscopy, cholestasis, liver biopsy, erythropoietic protoporphyria

## Abstract

Erythropoietic protoporphyria (EPP) is a rare inherited disorder of porphyrin metabolism that can cause liver damage and cholestatic hepatocellular failure. We report a case of EPP in a teenaged male who underwent liver biopsy for investigation of liver dysfunction of unknown cause. The diagnosis was not made until a re-biopsy approximately three years later, when the patient presented with recurrent skin lesions and elevated blood and urinary protoporphyrin levels. The liver biopsies contained brownish deposits that exhibited birefringence under polarized light and porphyrin fluorescence under fluorescence spectroscopy. EPP should be considered in young patients with unexplained liver dysfunction, skin symptoms, and seasonal changes in symptoms. Fluorescence spectroscopy of liver biopsy tissue can be a useful tool in the diagnosis of EPP.

## Introduction

Erythropoietic protoporphyria (EPP) is a rare inherited disorder of porphyrin metabolism caused by decreased activity of ferrochelatase (FECH), an enzyme that catalyzes the chelation of iron into protoporphyrin to form heme [1-4]. FECH deficiency is associated with increased concentrations of protoporphyrin in erythrocytes, plasma, skin, and liver [5]. Protoporphyrin accumulation in the skin causes photosensitivity, which is the chief symptom [1,3].

Accumulation of protoporphyrin in the liver leads to liver damage, and progressive hepatocellular disease ultimately leads to cholestatic hepatocellular failure, which often has an acute onset and a rapidly progressive, irreversible course [5]. Up to 35% of patients with EPP have mildly abnormal biochemical tests of liver function. Liver failure caused by the hepatotoxic action of protoporphyrin complicates about 2% of cases, in whom liver transplantation is often required [6,7]. Because of its often subtle and unspecific clinical symptoms, the disorder can be overlooked for years, resulting in a major delay between disease onset and diagnosis, and in a high degree of suffering in affected individuals [8]. Early diagnosis is therefore desirable.

We report a case of EPP in a teenage male who had a liver biopsy for liver dysfunction of unknown cause, and in whom EPP was not diagnosed until re-biopsy approximately three years later.

## Case presentation

**Figure 1:**
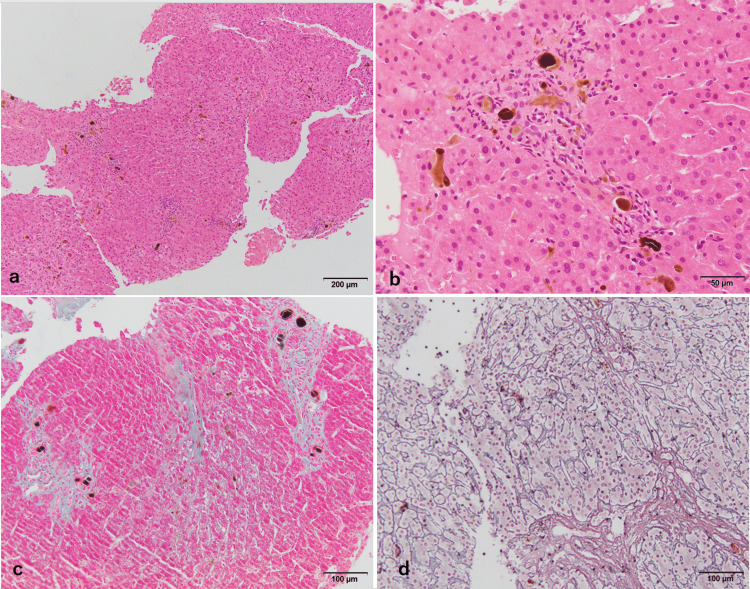
Histopathological images of the first biopsy at age 15 years. a, b: Hematoxylin and eosin stain. Mild lymphocytic infiltration is seen mainly in the portal region, and brownish deposits are located mainly in the portal area. c: Azan staining. There is mild fibrotic enlargement of the portal area. d: Gitter staining. Hepatic cords are mildly distorted with mild fibrous extension.

A 15-year-old male initially presented to his primary physician on December 2018 with chief complaints of fever and vomiting. Liver dysfunction was identified, and a liver biopsy was performed. Laboratory data at the time of the liver biopsy are shown in Table 1. Hepatitis virus infection (HBV and HCV) was not detected. No special note was found in the drug history. Histopathologically, marked brown pigments were observed in the portal area and hepatic parenchyma, with a fibrous extension of the portal area (Figure 1). The patient was diagnosed with severe cholestasis based on chronic inflammation of unknown etiology.

**Table 1:** Laboratory data at the first biopsy at age 15 years WBC: white blood cell count, RBC: red blood cell count, Hb: hemoglobin, PLT: platelet count, BUN: blood urea nitrogen, Cre: creatinine, T-bil: total bilirubin, D-bil: direct bilirubin, AST: aspartate aminotransferase, ALT: alanine aminotransferase, γ-GT: γ-glutamyl transpeptidase, ALP: alkaline phosphatase, LDH: lactate dehydrogenase; NH_3_: ammonia, Na: sodium, K: potassium, Cl: chlorine; Fe: iron

Lab	Value	Reference Range
WBC	6.11	3.6-9.0X10³/μl
Neutrophil	76.6	40-69%
Lymphocyte	16.9	26-46%
RBC	5.55	3.87-5.25X10⁶/μl
Hb	14.1	12.6-16.5g/dl
PLT	350	138-309X10³/μl
BUN	12.9	7-24mg/dl
Cre	0.62	≤1.0mg/dl
Na	137	135-147mmol/L
K	4.6	3.3-4.8mmol/L
Cl	97	98-108mmol/L
T-bil	9.66	0.2-1.2mg/dl
D-bil	6.97	0-0.3mg/dl
AST	251	≤ 30U/l
ALT	425	≤ 30U/l
γ-GT	354	≤ 50U/l
ALP	892	38-113U/l
LDH	217	119-229U/l
NH_3_	41	30-86μg/dl
Ferritin	62.3	20-250ng/ml
Fe	96	54-200μg/dl

**Figure 2:**
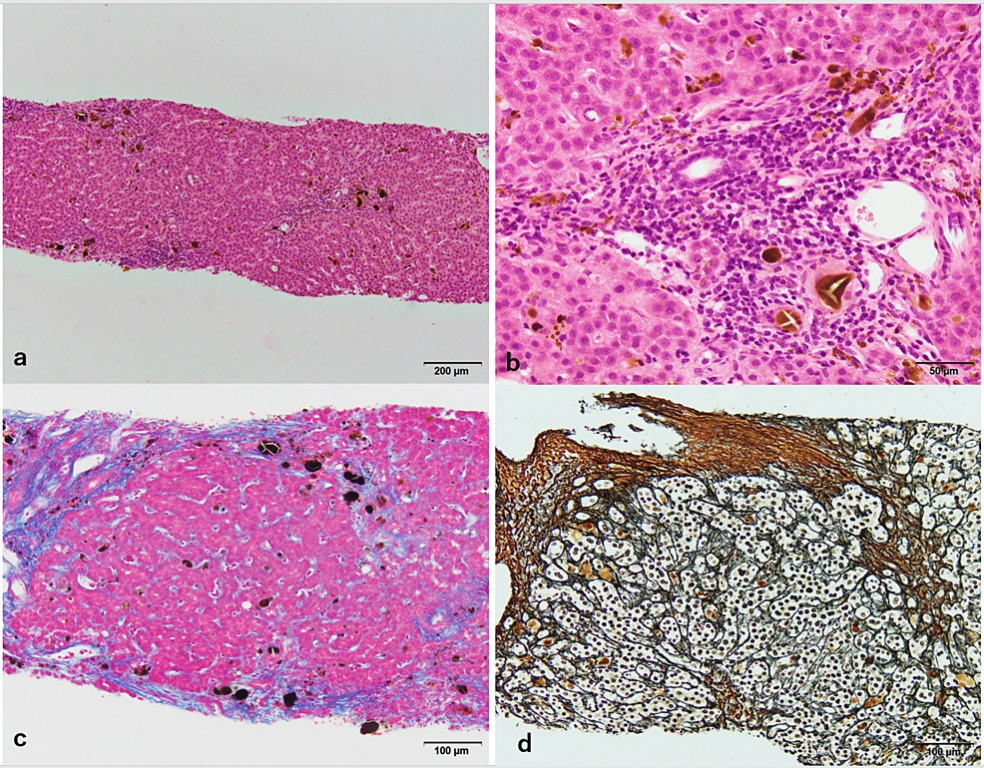
Histopathological images of the re-biopsy at age 18 years. a, b: Hematoxylin and eosin stain. Lymphocytic infiltration is seen mainly in the portal region, and brownish deposits are located in the fibrous septa as well as the hepatic parenchyma and sinusoids. c: Azan staining. Patchy fibrotic enlargement and bridging fibrosis are observed. d: Gitter staining. Hepatic cords are mildly distorted and partially surrounded by fibrous septa.

**Figure 3:**
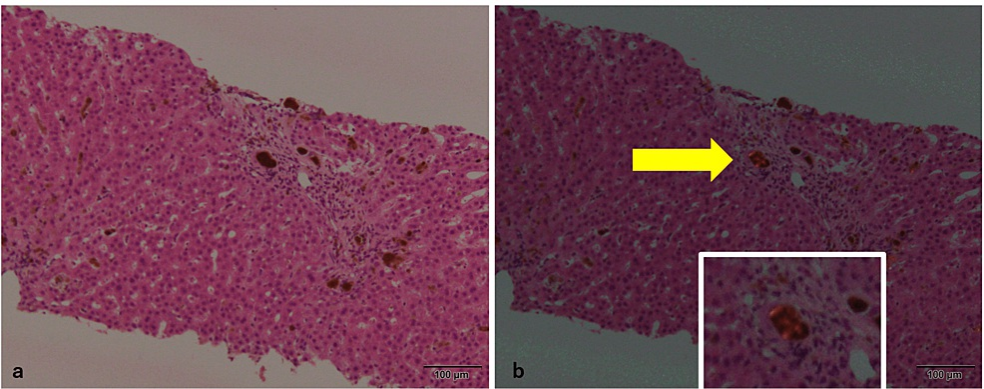
Polarized light observation of the re-biopsy specimen a: Hematoxylin and eosin stain. b: Under observation by polarized light, reddish-colored materials are seen at the periphery of the brown deposits (arrow).

In June 2020, he presented to his primary physician again complaining of upper abdominal pain, and liver dysfunction was found once more. He was then referred to our hospital for further investigations. On examination, it was found that he had been suffering from recurrent skin lesions on exposed areas since around 2016, especially in the summer. EPP was suspected, and another liver biopsy was performed in August 2021. Laboratory data obtained at that time are listed in Table 2. Histopathologically, the hepatic lobular architecture was moderately distorted. Mild lymphocytic infiltration and fibrosis were seen in the portal area. The degree of fibrosis was more prominent than in the first liver biopsy and fibrous septa extended from the portal area, showing bridging fibrosis in places. Dark brown to light brown deposits were observed at the margins of the fibrous septa and within the liver parenchyma. Some of these were located in the sinusoids, mainly inside Kupffer cells (Figure 2). Iron stain and copper stain (use orcein stain) were not found. Some of the brown deposits showed weak polarization and a reddish color in polarized light observation (Figure 3).

**Table 2:** Laboratory data at re-biopsy at age 18 years WBC: White Blood Cell count, RBC: Red Blood Cell count, Hb: Hemoglobin, PLT: Platelet count, BUN: blood urea nitrogen, Cre: creatinine, T-bil: total bilirubin, D-bil: direct bilirubin, AST: aspartate aminotransferase, ALT: alanine aminotransferase, γ-GT: γ-glutamyl transpeptidase, ALP: alkaline phosphatase, LDH: lactate dehydrogenase

Lab	Value	Reference Range
WBC	5	3.6-9.0X10³/μl
Neutrophil	62	40-69%
Lymphocyte	29	26-46%
RBC	4.95	3.87-5.25X10⁶/μl
Hb	13.5	12.6-16.5g/dl
PLT	171	138-309X10³/μl
BUN	14	7-24mg/dl
Cre	0.65	≤1.0mg/dl
Na	138	135-147mmol/L
K	4	3.3-4.8mmol/L
Cl	102	98-108mmol/L
T-bil	9.5	0.2-1.2mg/dl
D-bil	6.7	0-0.3mg/dl
AST	80	≤ 30U/l
ALT	99	≤ 30U/l
γ-GT	177	≤ 50U/l
ALP	216	38-113U/l
LDH	203	119-229U/l
NH3	37	30-86μg/dl
Ferritin	52	20-250ng/ml
Fe	161	54-200μg/dl
Protoporphyrin (RBC)	4143	30-80μg/dl RBC
Uroporphyrin (urinary)	80	2-25μg/gcre
Coproporphyrin(urinary)	840	8-168μg/gcre

**Figure 4:**
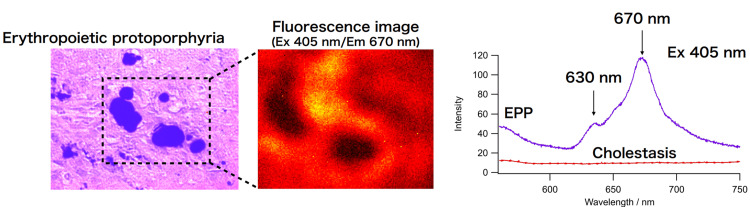
Fluorescence spectroscopy with 405 nm excitation light Obtained positive porphyria fluorescence at around 670 nm. Porphyrin was located at the periphery of the dark brown bile pigments

We re-examined the first biopsy specimen and found the same brownish materials, which differed from the original bile pigmentation. However, the findings of polarized light observation were unclear. We then attempted to evaluate the specimens using fluorescence spectroscopy with 405 nm excitation light, and obtained positive porphyria fluorescence at around 670 nm. Porphyrin was located at the periphery of the dark brown bile pigments (Figure 4)

Further clinical examination revealed elevated blood protoporphyrin and urinary uroporphyrin, and genetic testing showed an abnormality in the *FECH* gene. Researching the family history, it was found that his mother and elder brother also had photosensitivity. And they also had the same abnormality in the *FECH* gene. Finally, the patient was diagnosed with EPP. 

## Discussion

In EPP, brownish deposits in the bile canaliculi and Kupffer cells within the liver tissue exhibit a characteristic birefringence (Maltese cross pattern) under polarized light [4,9,10]. However, these deposits may be overlooked or misdiagnosed as cholestasis due to their similar appearances, and also because observation under polarized light is not routinely performed. These make pathological diagnosis difficult especially for pathologists without any experience with EPP. Therefore, porphyria should be considered a potential cause of liver damage in young patients, especially if photosensitivity is present.

In the event that birefringence is difficult to detect with polarized light microscopy, fluorescence microscopy may enable the diagnosis. The brownish deposits seen in porphyria fluoresce at around 670 nm when excited by 405 nm light can help distinguish simple biliary stasis from porphyrin deposits associated with biliary stasis.

In this case, EPP was overlooked at the first biopsy, and almost three years passed without appropriate treatment. As a result, the liver fibrosis degree was elevated. In review, this case was liver dysfunction of the young male without any special note such as virus infection and drug history, and EPP should have been included in the differential diagnosis. And if it had been known that protoporphyrins could be identified by fluorescence microscopy, diagnosis of EPP was possible at the first biopsy.

## Conclusions

We presented a case of EPP in a young male who was initially misdiagnosed but was correctly diagnosed three years later using polarized light observation and fluorescence microscopy. To avoid a delayed diagnosis, EPP should be considered in the differential diagnosis of unexplained liver dysfunction in young patients, and both polarized light observation and fluorescence microscopy should be used to confirm the presence or absence of protoporphyrin deposits.
